# Attitudes toward mathematics/statistics, anxiety, self-efficacy and academic performance: an artificial neural network

**DOI:** 10.3389/fpsyg.2023.1214892

**Published:** 2023-07-10

**Authors:** Juan Manuel Hernández de la Hera, Francisco Manuel Morales-Rodríguez, José Pablo Rodríguez-Gobiet, Juan Pedro Martínez-Ramón

**Affiliations:** ^1^Department of Evolutionary and Educational Psychology, Faculty of Education, Cartuja Campus, University of Granada, Granada, Spain; ^2^Department of Evolutionary and Educational Psychology, Faculty of Psychology, Cartuja Campus, University of Granada, Granada, Spain; ^3^Department of Evolutionary and Educational Psychology, Faculty of Psychology and Speech Therapy, Campus Regional Excellence Mare Nostrum, University of Murcia, Murcia, Spain

**Keywords:** attitudes, anxiety, educational psychology, mathematics, artificial neural network, university student

## Abstract

Mathematics and statistical skills are crucial to daily life. However, many students found mathematics difficult to learn and understand. This research aimed to find relationships between mathematics and statistical attitudes and emotional dimensions, such as anxiety or self-efficacy. The sample consisted of two groups: the first group was formed by 276 Spanish students (75.7% female with an average age of 19.92 years) from different degrees at the University of Granada and the second one by agroup of 19 secondary school students from of a Secondary School in Granada, Spain (57.9% male students between 14 and 16 years of age from a public school). The instruments applied were a scale of attitude toward mathematics, a scale of attitude toward statistics, a scale to assess mathematical anxiety, and a scale to assess self-efficacy. An artificial neural network for the backpropagation algorithm was designed using dependent variable. The results showed a negative impact of anxiety on those attitudes, while self-efficacy had a positive impact on those mentioned attitudes. Therefore, emotional education is important in the well-being, and teaching in mathematics. The usefulness of the innovative neural network analysis in predicting the constructs evaluated in this study can be highlighted.

## 1. Introduction

When we talk about attitudes toward mathematics we are somehow referring to the affective component of the construct (Geisler et al., 2023) that Emmioğlu and Çapa-Aydın (2012, p. 95) define as a “multidimensional construct that stands for students’ learned predispositions to respond positively or negatively with regard to statistics.” It is necessary not to neglect this non-cognitive component so relevant to the teaching/learning process in Mathematics and Statistics (García-Suárez et al., 2023; Spencer et al., 2023). The affective dimension of competencies such as emotional competence is part of the curriculum to be addressed in the teaching/learning processes of mathematics. Therefore, the evaluation of these attitudes is essential for academic achievement and performance improvement, since the affection or rejection toward mathematics has an impact on the interest and motivation toward mathematics, as indicated by Feregrino et al. (2021). Also within an affective-emotional dimension, the term self-efficacy refers to “beliefs about their ability to perform statistical/mathematical tasks (Spencer, 2023, p. 1), and the term mathematical anxiety refers to “feelings of tension and discomfort that might prevent someone from carrying out his or her actual capability in mathematical problems” (Ashcraft, cited in Özcan and Eren Gümüş, 2019, p. 119) and this may be related to a higher frequency of negative attitudes toward mathematics (García-Suárez et al., 2023) with the consequences this may have for their learning and performance and for the specific process for the instruction and learning of mathematics and statistics. By assessing these constructs we focus on non-cognitive aspects, which have sometimes been given less importance, and may be relevant to achieve meaningful learning in mathematics and improve its teaching (Ávila-Toscano et al., 2020; Sánchez et al., 2022; García-Suárez et al., 2023).

Regarding gender, Galende et al. (2020) reported more positive attitudes toward mathematics in males compared to females. Paechter et al. (2017) found higher levels of math anxiety in females compared to males. Likewise, Ryan et al. (2022) found statistically significant gender differences in the variables of math anxiety, math self-efficacy and perseverance; specifically, it was males who presented higher levels of mathematical self-efficacy and self-concept compared to females, while females presented higher levels of mathematical anxiety compared to males. It should be noted that this has psychoeducational implications for the teaching/learning process since, as indicated by Ryan et al. (2022), women are more concerned about possible difficulties in learning mathematics and lower mathematics achievement than men. In the same line, Rončević Zubković et al. (2021) evaluated mathematical anxiety, mathematical self-concept and learning strategies and mathematical performance in a sample formed by 2,749 Croatian student body, 56% being girls, with a mean age of 14.58 years finding a less positive mathematical self-concept in girls compared to boys and a higher use of so-called learning strategies; girls were also found to have higher levels of mathematics anxiety compared to boys. It was also found that the older the age of the transition to school, the greater the lack of motivation, the positive evaluation of mathematics and the lower the academic performance.

Studies that have previously analyzed the relationship between the constructs included in the present study are shown below. Regarding the relationships between attitudes toward mathematics and the constructs of anxiety and other emotional factors, a recent study (Xiao and Sun, 2021) examined affective-motivational factors such as attitudes and interest toward mathematics, mathematical self-concept and mathematical anxiety in a sample of U.S. students, 51% of whom were male. They found different motivational and affective profiles, being the students with a higher level of motivation and lower level of mathematical anxiety those with higher persistence and mathematical performance. In this study, the importance of this type of results is raised so that teachers can also attend to motivational diversity with individualized instruction. In the same vein, Geisler et al. (2023) indicate that there is some evidence of relationships between the individual interest factor of the construct of attitudes toward mathematics with academic performance, but that more research is needed as the results are not entirely conclusive as there are studies (e.g., Rach and Heinze, 2017) even contradictory that find no relationships between the interest factor and academic performance. Among previously studied theoretical models, those proposed by Paechter et al. (2017), in which other variables such as procrastination are included, stand out. The following instruments were used in this study: the German adaptations of the Statistics Anxiety Rating Scale (STARS; Cruise et al., 1985; Papousek et al., 2012), Revised Mathematics Anxiety Ratings Scale (R-MARS; Baloğlu and Zelhart, 2007), State–Trait-Anxiety Inventory (STAI, German version; Laux et al., 1981) and Procrastination Assessment Scale-Students (PASS; O’Callaghan et al., 2009; Macher et al., 2012). The researchers reached the following conclusions: female students presented higher levels of math anxiety; those with a higher propensity to experience anxiety in general, also experienced higher levels of math anxiety; better grades in mathematics implied lower levels of math anxiety; better grades in mathematics implied surprisingly higher levels of statistical anxiety; math anxiety was directly related to statistical anxiety; males were more prone to procrastination; low grades were related to higher levels of procrastination; and statistical anxiety was related to procrastination, among others. Another model to be highlighted is that proposed by Weidinger et al. (2017), who suggested time interval models were related to motivation and grades. In this study, whose sample consisted of 542 German elementary school students, the instruments were applied over 2 years (from Grades 2 to 4), and the following conclusions were obtained: contrary to what might be expected, the findings in this field showed that negative feedback does not always lead to a decrease in internal or intrinsic motivation. This calls into question the widely held view that the idea of thinking of less proficient, as reflected in poor grades, weakens students’ intrinsic motivation. Regarding mathematics anxiety, it is worth mentioning that Orbach et al. (2019) investigated mathematics anxiety by comparing the trait components of mathematics anxiety with real-time assessments of situational anxiety responses among children. In that study, different aspects were measured, such as components of math anxiety, self-assessment of math skills, attitudes toward mathematics, learning motivation, mathematics performance, social anxiety, and intelligence of 1.179 4th and 5th grade students. A negative correlation was observed between a component of mathematics anxiety and academic performance. Lim and Chapman (2015) studied the effects of using mathematics history in mathematics instruction on levels of mathematics anxiety, attitudes, and motivation in grade 11. Four classes from a school in Singapore participated in this experiment. The experimental group of 51 students and control group of 52 students comprised two classes each. The results indicated that the use of stories as a tool for teaching mathematics had desirable outcomes. Noteworthy are the studies conducted by Abín et al. (2020) and Suren and Kandemir (2020) on their effects on performance. Suren and Kandemir (2020) administered the following instruments to a sample of 777 grade 8 students from a province in the Aegea region of Turkey: Mathematical Motivation Scale (MMS) and Mathematics Anxiety Scale for Elementary School Students (MASESS). According to that research, the levels of anxiety and motivation may have a positive relationship when it comes down Mathematics. In fact, anxiety was found to predict performance at a higher level, followed by motivation. The same year, in a study by Abín et al. (2020), a large sample of 2.365 Spanish students from four secondary school grades (12–16 years) participated. In that research, information was collected on students’ intellectual abilities, perceived competence in mathematics, perceived usefulness of mathematics, mathematics anxiety, causal attributions, and mathematics achievement. They found differences based on sex, but no significant effects of affective or motivational variables. Özcan and Eren Gümüş (2019) investigated the direct and indirect effects of several noncognitive constructs, such as mathematical self-efficacy, mathematical anxiety, and metacognitive expertise, on mathematical problem solving in high school students. The participants were 517 Turkish grade 7 students. They found an effect of mathematical anxiety and academic motivation, with self-efficacy as a mediating variable in solving mathematical problems. Likewise, the effects of metacognitive experience mediated the variables of mathematical anxiety, self-efficacy, and motivation. These metacognitive experiences also influenced the resolution of mathematical problems. Also, Li et al. (2021) conducted a meta-analysis to determine the relationship between mathematics anxiety and students’ motivation to learn mathematics and found a moderate negative correlation between students’ mathematics motivation and mathematics anxiety. Regarding the relationships between attitudes toward mathematics and the variable self-efficacy, there is also previous research (Czocher et al., 2020) that found that academic self-efficacy was one of the relevant predictor variables of motivation and persistence in STEAM (science, technology, engineering, art, and mathematics) careers. Czocher et al. (2020) proposed the training of self-efficacy and competence through mathematical modeling in university students, indicating the importance of an intervention to improve the construction of such self-efficacy. Along the same lines, another recent study highlights the importance of mathematics attitudes such as academic interest and self-concept and aspects such as degree of satisfaction and perceived achievement given their influence on the dropout rate during the first university years (Geisler et al., 2023). Czocher et al. (2020) found in first-year undergraduate students at a German university in some mathematics major (mathematics program and teacher education program) aged 17–33 years, using a gender-balanced sample, that attitudes toward mathematics such as mathematical interest and mathematical self-concept correlated inversely with the risk of academic dropout; to be specific, satisfaction was a mediating variable for both academic interest and academic self-concept. Another study assessed attitudes toward statistics, statistical self-efficacy, task value and task effort using a convenience sample of 189 students, mostly second year engineering students, more than half white, 76.2% male (Spencer et al., 2023). They also found that self-efficacy and affect were predictive variables of overall course grade, although not of course retention.

Focusing on all the indicated constructs, another previous research analyzed attitudes toward mathematics and other aspects related to the emotional dimension such as perceived self-efficacy and math anxiety in a sample of 304 Irish Secondary Education students, 60.2% male (Ryan et al., 2022). These authors considered it essential to start assessing motivation toward mathematics in this period, especially in view of the implementation of a new curriculum in this area in that country. They found in the students participating in this study positive self-confidence and also high levels of commitment and motivation in mathematics even after 1 year of instruction. Özcan and Eren Gümüş (2019) using structural equation modeling found in a study involving Turkish secondary school students, effects of math anxiety and motivation on perceived self-efficacy and metacognitive experience which at the same time impact on the variable academic achievement. Another work also highlighted the importance of interventions aimed at improving self-efficacy and self-regulated learning that has at the same time effects on the non-cognitive factor, that is, on the emotional dimension (enjoyment, achievement, and mathematical anxiety) of the student body, although they found no statistically significant differences in the variable academic performance in mathematics after such intervention (Gamlem et al., 2019). Likewise, another study conducted in 2022 by Rovan et al. (2022) examined the extent to which prior motivational beliefs, such as self-efficacy and subjective value, in a sample consisting of 237 future first-year elementary school teachers are related to their prior experiences related to mathematical learning and math anxiety. They found that prior experience was a predictor variable of mathematics self-efficacy and subjective value. The variable of prior motivational beliefs was also a predictor variable of mathematics anxiety and somewhat in the participation in the teaching/learning process in the prospective teachers in the previous study.

More recently, García-Suárez et al. (2023) also analyzed the affective-emotional dimension, specifically mathematical anxiety and the level of self-confidence, in a sample of 174 first-year, undergraduate engineering students from one of the centers of the University Network of the University of Guadalajara (Mexico). They found, in general terms, a tendency toward a moderate level of math anxiety, but they also found very high levels of anxiety in some students. They also found statistically significant negative correlations between the levels of math anxiety and the degree of self-confidence; The authors show that even in mathematics-related careers, these students may present levels of anxiety that are important to evaluate with a view to improving the teaching/learning process.

Regarding recent studies carried out in Spain, a study evaluated attitudes toward mathematics in a sample of 81 university students belonging to the Primary Education degree, finding (Garcia-Manrubia et al., 2022): (a) values considered adequate for the motivation and confidence factors; (b) values considered medium for the low interest factor; and (c) medium-low values for the liking and anxiety factors. Likewise, this study highlighted the need, with a view to the training of future teachers, to further deepen the scoring of these constructs in future research, especially considering the socio-affective domain. Also using a sample of Spanish students, it is worth mentioning the study by Hossein-Mohand and Hossein-Mohand (2023), which aimed to analyze the effect of motivation on the perception of mathematics in high school students. The instrument consisted of 135 items with six dimensions and 31 indicators, following the procedure of Rosenbluth et al. (2016). This instrument was applied to 2039 students from various educational levels in the centers of the Autonomous City of Melilla. In this study, no correlations were found between sex, educational level, and motivation. The researchers reached the following conclusions: motivation levels could be influencing other study behaviors; there were no significant differences between sex and educational level, study time or motivation; there were no differences between motivation and mathematical learning; and there were certain differences between women and men in the teaching variable.

Several instruments have been applied for the evaluation of the above variables, which are as follows: Scale of attitude toward mathematics (Auzmendi, 1992), Scale of attitude toward statistics (Auzmendi, 1992; Darias Morales, 2000), Scale for Assessing Math Anxiety in Secondary education (SAMAS) (Yáñez-Marquina and Villardón-Gallego, 2017) and General Self-Efficacy Scale (EAG) (Baessler and Schwarzer, 1996; Espada et al., 2012). Other instruments to consider are, for example, the Math anxiety subscale of the Fennema-Sherman Mathematics Attitude Scale (MAS; Fennema and Sherman, 1976), which was considered for application, although it was finally discarded because of the greater suitability of the previous instrument for math anxiety, as it has already been applied and adapted to the context of Spanish education and society, showing excellent results. The scale of attitude toward mathematics (Auzmendi, 1992) and scale of attitude toward statistics (Auzmendi, 1992) are brief self-reports in a 25-item format used to assess the relevant dimensions of attitudes toward mathematics and statistics. Moreover, these instruments have been applied in the Spanish context, both in primary and secondary education and at post-compulsory levels, which is an indicator of the suitability and effectiveness of both tools. Other instruments selected, such as the mathematical anxiety and self-perceived efficacy instruments, were reduced versions that have been widely administered in the Spanish context and cover the most relevant dimensions of these constructs. Specifically, in the case of mathematics, it is especially relevant to understand the existing attitudes and social and emotional competencies of students in this area. Another noteworthy study is that of Ren et al. (2016), which used the previously adapted Fennema-Sherman scale of mathematical attitudes for primary school teachers. This study was divided into three phases: the first pilot phase to check whether the modifications made were adequate; the second phase with 225 teachers as a sample for a subsequent factor analysis; and the third phase for an invariance analysis with 171 teachers as a sample. The overall results suggested that the revised scale could be used by researchers and program evaluators to reliably measure attitudes toward mathematics among elementary school teachers and could be a valuable tool for assessing the effectiveness of professional development. Regarding the variables of attitudes toward statistics and mathematics, Flores and Auzmendi (2015) verified the suitability of the variables described above for attitudes toward mathematics. In Darias Morales’s (2000) study, the same was done for the variables with respect to attitudes toward statistics. Regarding mathematical anxiety, the model used, with duly justified variables, appeared in the study of Yáñez-Marquina and Villardón-Gallego (2017), in which the influences of other scales on its elaboration were also discussed. The Generalized Self-Efficacy Scale (GSES) has been considered (Baessler and Schwarzer, 1996; Espada et al., 2012), and validated by Espada et al. in Spain.

It is worth pointing out the need to continue to study in depth the affective domain related to the mathematics curriculum, considering also that there are still no studies in which the relationships between these constructs are studied by means of the design of artificial neural networks. Precisely, there are already studies that have been demonstrating the usefulness of this type of analysis given the numerous applications that can be derived to see how these variables influence the teaching/learning process (Hwang et al., 2020; Martínez-Ramón et al., 2021) and which would also be appropriate, in this case, in Mathematics due to their potential predictive value for the so-called Mathematics Learning Difficulties (MALD) in students and with the help of the “intelligent tutor” from the teaching environment (Hwang et al., 2020).

Having said that, the present project seeks to study different variables related to students’ attitudes toward mathematics and/or statistics, and find relationships between these and other variables, such as academic performance or anxiety generated in different situations involving mathematics and/or statistics. The variables to be addressed are (a) attitudes toward mathematics (disaggregated into anxiety -levels of alteration or nervousness-, agreeableness -levels of affinity with mathematical activity-, usefulness -level of perceived usefulness of mathematics-, motivation -level of motivation generated by mathematics-, and confidence -level of hope generated by mathematics-); (b) attitudes toward statistics (disaggregated into security -related to aspects of anxiety, but also with the perception of security/insecurity with respect to the ability to execute statistical problems-, importance -with certain connotations of satisfaction and valuation of the subject-, usefulness -measures the productivity or benefits that statistics can offer-, and desire to know -including aspects of motivation toward knowledge, although also related to aspects of usefulness-); (c) mathematics anxiety (disaggregated into anxiety in everyday mathematics -anxiety generated by everyday activities involving basic mathematics-, mathematical learning -anxiety generated by the activities performed throughout the learning process in mathematics-, and mathematics tests -anxiety generated by facing and taking mathematics tests-); (c) self-perceived efficiency (self-perceived degree of performance in dealing with various situations); and (d) sociodemographic data (sex, age, grade, degree, and average grade).

The overall objective of the present study was to analyze the possible causes of student demotivation toward mathematics, find relationships between these causes and academic performance, and propose a learning situation/teaching unit based on statistics/probability as a response to these possible relationships and/or causes. The specific objectives were as follows: (1) to study the differences in these variables between different groups according to age and sex; (2) to examine the relationship between mathematics and statistics attitudes and study variables; (3) to study differences in mathematical anxiety before and after a mathematics exam; and (4) to design an artificial neural network (ANN) capable of predicting academic performance on the basis of scores on the variables math anxiety, self-efficacy and sociodemographic variables of the study.

The hypotheses of the present work were: (h1) we expected to find statistically significant gender differences in the levels of mathematical anxiety and self-efficacy; (h2) we expected to find associations between attitudes toward mathematics and statistics and the variables of mathematics anxiety and perceived self-efficacy -in particular we expected to find a direct negative relationship between academic performance and variables related to mathematics anxiety, in contrast, a positive direct relationship was expected between academic achievement and variables related to attitudes toward mathematics, statistics, and self-efficacy-; (h3) to study the levels of math anxiety before and after a math test, higher levels of anxiety were expected before taking the exam; (h4) it was expected that the levels of math anxiety, self-efficacy and the sociodemographic variables of the study may contributed to the predictive capacity of the artificial neural network of the variable academic performance in mathematics.

## 2. Methods

### 2.1. Design

The ethical principles of the Declaration of Helsinki were considered and an *ex post facto* cross-sectional design was used. After obtaining approval from the Ethics Committee and reporting on the project, self-reported questionnaires with Likert-type responses were administered to participants.

### 2.2. Participants

The study protocol was approved by the Ethics Committee of the University of Granada (Granada, Spain; 3,376/CEIH/2023). Full-time students between the ages of 12 and 58 years were included. Based on the way of selection of individuals, the sample could be catalogued as a convenience sample. Since there was no missing data, technics meant for this purpose were no needed. The sample comprised 276 Spanish students from different degrees and levels, including 209 females (75.7%) and 67 males (24.3%). The average age of female participants was 19.92 years, whereas that of male participants was 20.07. The female participants were aged between 18 and 59 years, whereas the male participants were between 18 and 45 years. For the study of mathematical anxiety before and after a mathematics exam, the sample comprised 19 secondary education students, consisting of eight females (42.1%) and 11 males (57.9%). The average age of female participants was 14 years, whereas that of male participants was 14.73 years. The female participants were all 14 years old, whereas the male participants were between 14 and 16 years old.

### 2.3. Procedure

The instruments were collectively administered in classrooms using an online link. The collaborating faculty reported the purpose of the research and its confidentiality in the presence of the members of the research team. Doubts have also been raised in this regard. The secondary school students were contacted by one of the teachers. Thereafter, the project was explained, and data and instruments were administered in the classroom. The teachers were able to clarify any doubts at all times. Data were collected between March and April 2023. The study was voluntary, confidential, and anonymous.

### 2.4. Measures

*Scale of attitude toward mathematics* (Auzmendi, 1992) assesses the dimensions of mathematics. This scale evaluates five different aspects, namely anxiety, pleasantness, utility, motivation, and confidence, using 25 different items. These items are evaluated on a continuous response scale ranging from 1 (strongly disagree) to 5 (strongly agree). The reliability analyses showed acceptable internal consistency (*ɑ* = 0.914).

*Scale of attitude toward statistics* (Auzmendi, 1992) assesses the dimensions of statistics. This scale evaluates four different aspects: security, importance, utility, and desire to know through 25 different items. These items are evaluated on a continuous response scale ranging from 1 (strongly disagree) to 5 (strongly agree). The reliability analyses showed acceptable internal consistency (*ɑ* = 0.9).

*Scale for assessing math anxiety in secondary education* (SAMAS) (Yáñez-Marquina and Villardón-Gallego, 2017). This self-report scale consists of 20 items with a Likert-type response format ranging from 0 (strongly disagree) to 10 (strongly agree). It comprises the following subscales: (a) *Anxiety regarding everyday mathematics*; (b) *Mathematical learning-anxiety*; and (c) *Math test anxiety*. Internal consistency for the subscale *Anxiety regarding everyday mathematics* was *ɑ* = 0.84; internal consistency for the subscale *Mathematical learning-anxiety* was *ɑ* = 0.86, and internal consistency for the subscale *Math test anxiety* was *ɑ* = 0.84. Examples of items for each of the factors, respectively, would be the following: (a) “I get nervous when calculating the total price of what I bought”; (b) “I get nervous whenever it is math’s turn”; (c) “I get more nervous during the math tests than during the exams of other subjects.”

*General self-efficacy scale* (EAG) (Baessler and Schwarzer, 1996; Espada et al., 2012). This instrument consists of 10 items with a 10-step Likert-type response format from 1 = “totally disagree” to 10 = “totally agree.” A total score is obtained in which the higher the score, the higher the level of self-efficacy. The internal consistency of the scale is 0.092. An example item is: “I can solve difficult problems if I try hard enough.”

*Sociodemographic questionnaire* on degree, course, sex, age, and average grade. *Ad hoc* preparation.

### 2.5. Statistical analysis

SPSS Windows software version 26 was used for statistical analysis. Descriptive analyses of this study are also presented (percentages, frequencies, means, and standard deviations). A *t*-test was used to compare means according to sex after verifying that the assumptions of homoscedasticity and normality were met. Stepwise regression analyses were performed. For non-parametric contrasts, the Kolmogorov–Smirnov (for comparing the distribution of two variables), Wilcoxon (for comparing the median value of two variables if they follow different not normal laws), Sign, and Mann–Whitney tests were applied when needed. The regression model included the dimensions of mathematics and statistics attitudes as dependent variables, and the dimensions of anxiety and perceived self-efficacy as independent variables (value of *p* lower than 0.05).

An artificial neural network for the backpropagation algorithm was designed using academic performance as the dependent variable. All ratings were transformed to a scale ranging from 0 to 10, and the 25th, 50th, and 75th percentiles were calculated to determine the Q1, Q2, Q3, and Q4 quartiles. Knowing the 75th percentile (Q1) in terms of performance, performance was recalculated in another variable so that two groups were dichotomized: a group of students with excellent performance (Q1) with a grade equal to or greater than 8.49 and the performance of the students who were at that level (Q2, Q3, and Q4). For the RNA signal, a fixed seed was established to manage randomness (314159265). Regarding the distribution of the cases, 68% (*n* = 187) were used for the training phase, 20.7% (*n* = 57) for the testing phase, and 11.3% (*n* = 31) for the training, testing, and backup phases, respectively. Regarding network information, the ANN consisted of a factor that was a sex-dichotomous qualitative variable and five-scale covariates (age, self-efficacy, Act_mat, Act_state, and math_anxiety) together with the bias node. The generated hidden layer was composed of two nodes and a bias. Finally, the VD was composed of two nodes (1 = performance of excellence, Q1, and 0 = academic performance, Q2, Q3, and Q4). No scaling was required for input-layer covariates. The activation function of the hidden layer in this research was a hyperbolic tangent. The output layer activation function was a softmax function and the output layer error function was cross-entropy.

## 3. Results

### 3.1. Descriptive results for the whole sample

The descriptive information for the variables mentioned above is shown in Table 1.

**Table 1:** Descriptive summary and internal consistency for mathematics and statistics attitudes, mathematical anxiety, and self-efficacy.

Variables	Minimum	Maximum	Mean	Standard deviation	Cronbach’s alpha
Attitudes toward mathematics					
Anxiety	9	45	27.190	8.257	0.746
Pleasure	4	20	9.450	3.907	0.717
Utility	6	30	15.890	4.690	0.717
Motivation	3	15	10.300	2.290	0.716
Trust	3	15	11.180	2.359	0.716
Attitudes toward statistics					
Security	11	55	32.890	8.768	0.730
Importance	6	30	14.860	4.720	0.709
Utility	5	24	14.660	3.460	0.712
Desire to know	5	24	16.500	3.341	0.714
Mathematical anxiety					
Anxiety regarding everyday mathematics	0	70	19.510	16.432	0.700
Mathematical learning-anxiety	0	80	32.420	21.290	0.743
Math test anxiety	0	50	29.650	13.826	0.703
Self-efficacy	12	40	29.590	4.845	0.718

### 3.2. Differences between sexes in levels of the variables mentioned above

**Figure 1:**
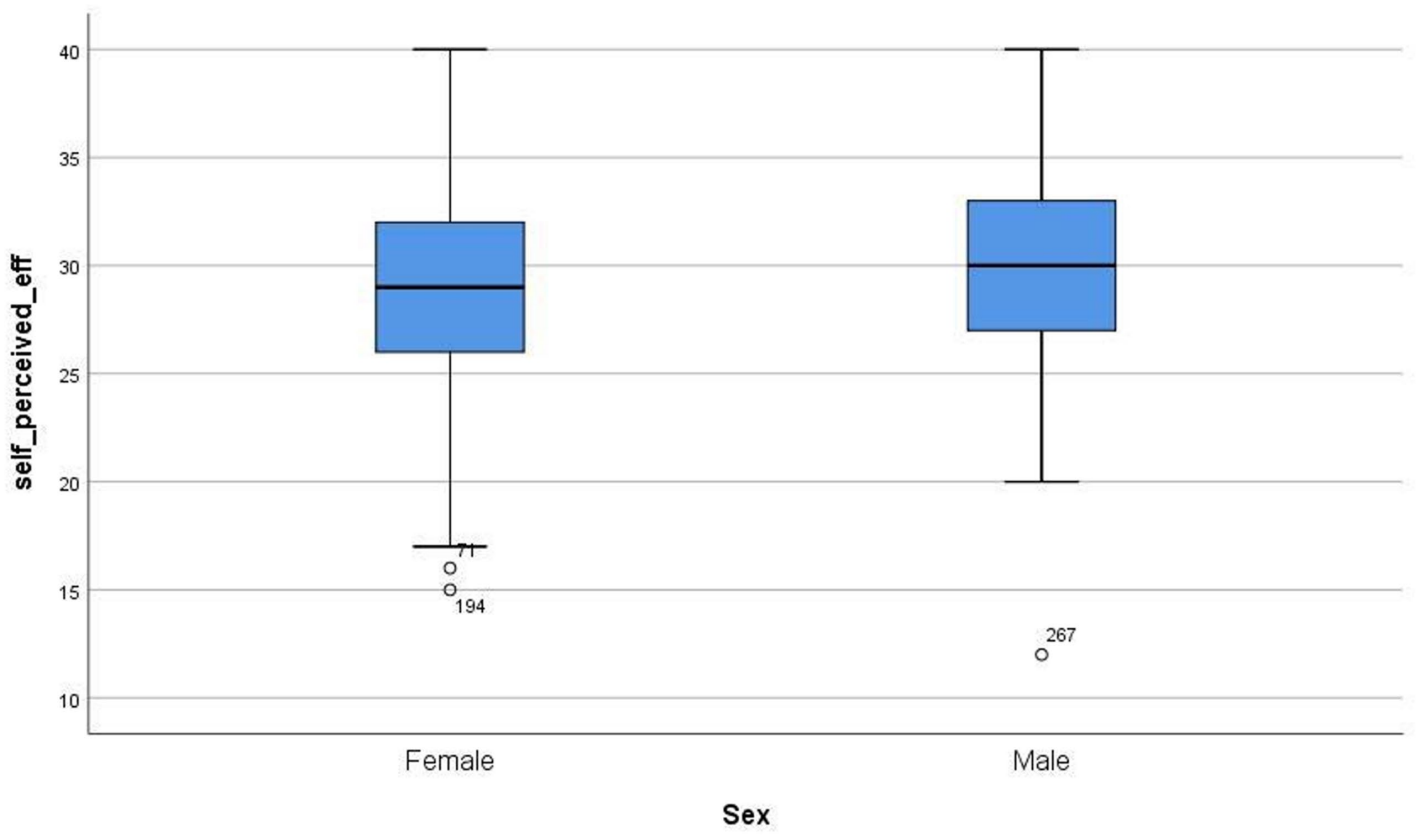
Differences between sexes in levels of self-efficiency.

**Figure 2:**
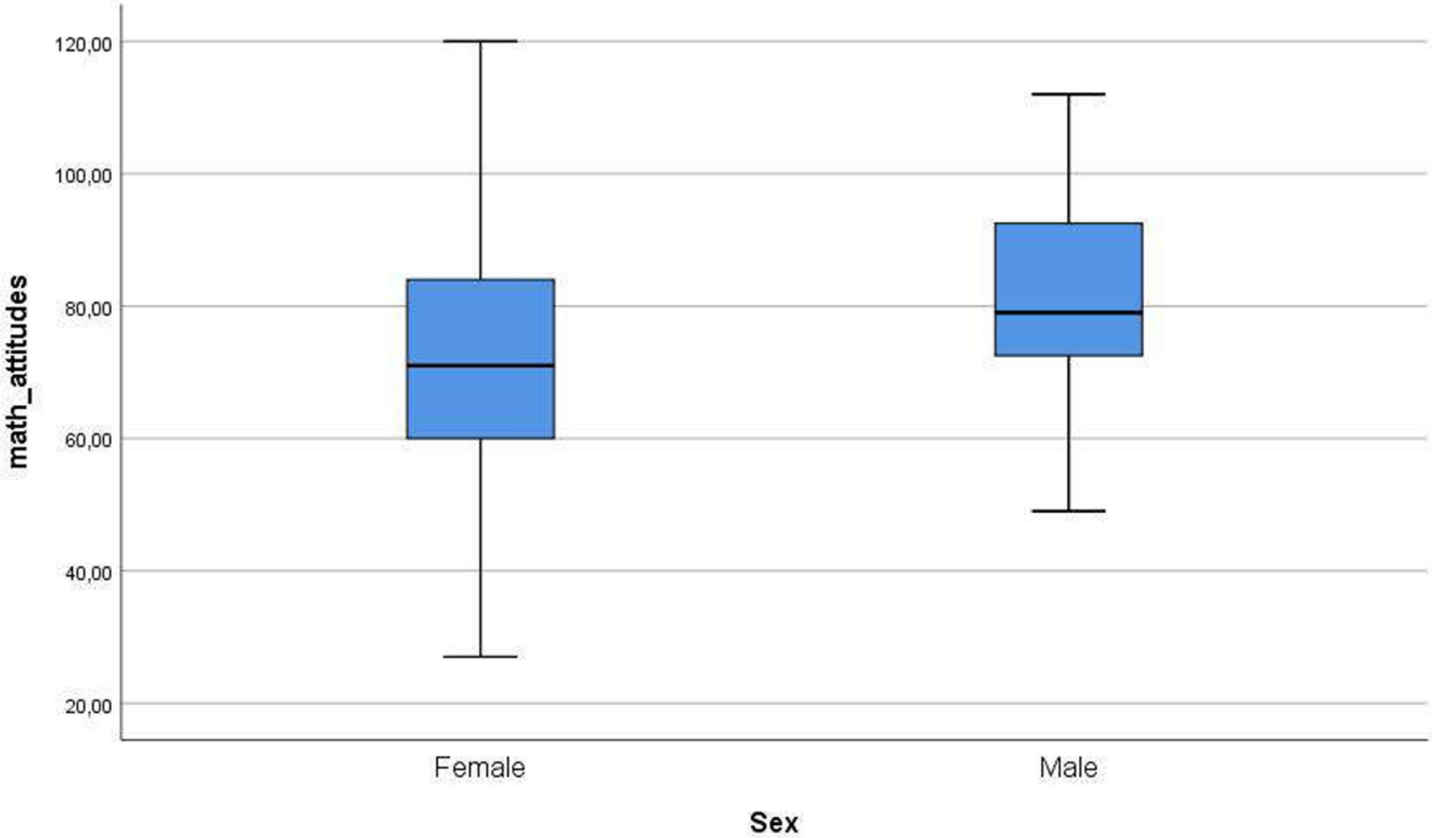
Differences between sexes in levels of mathematics attitudes.

**Figure 3:**
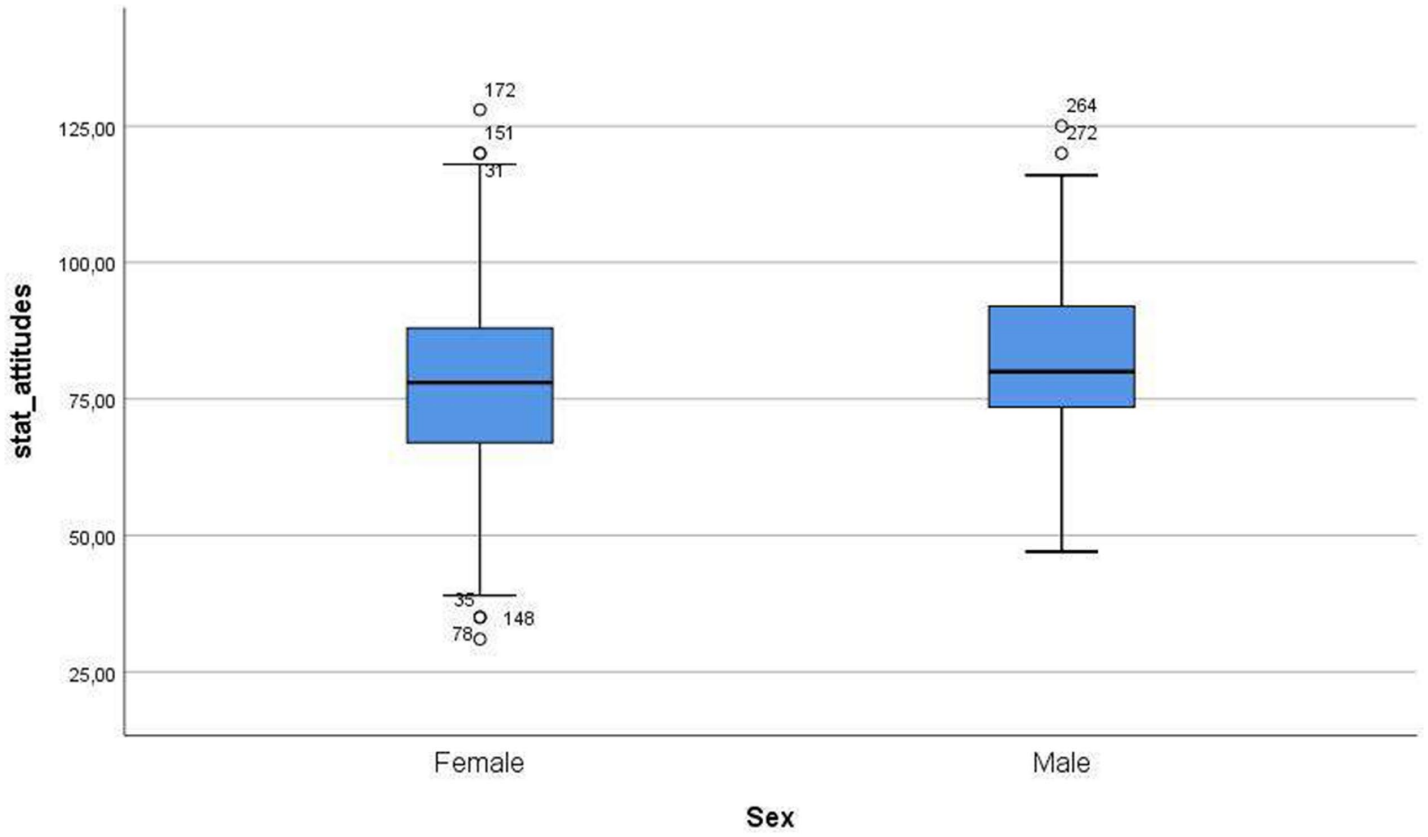
Differences between sexes in levels of statistics attitudes.

**Figure 4:**
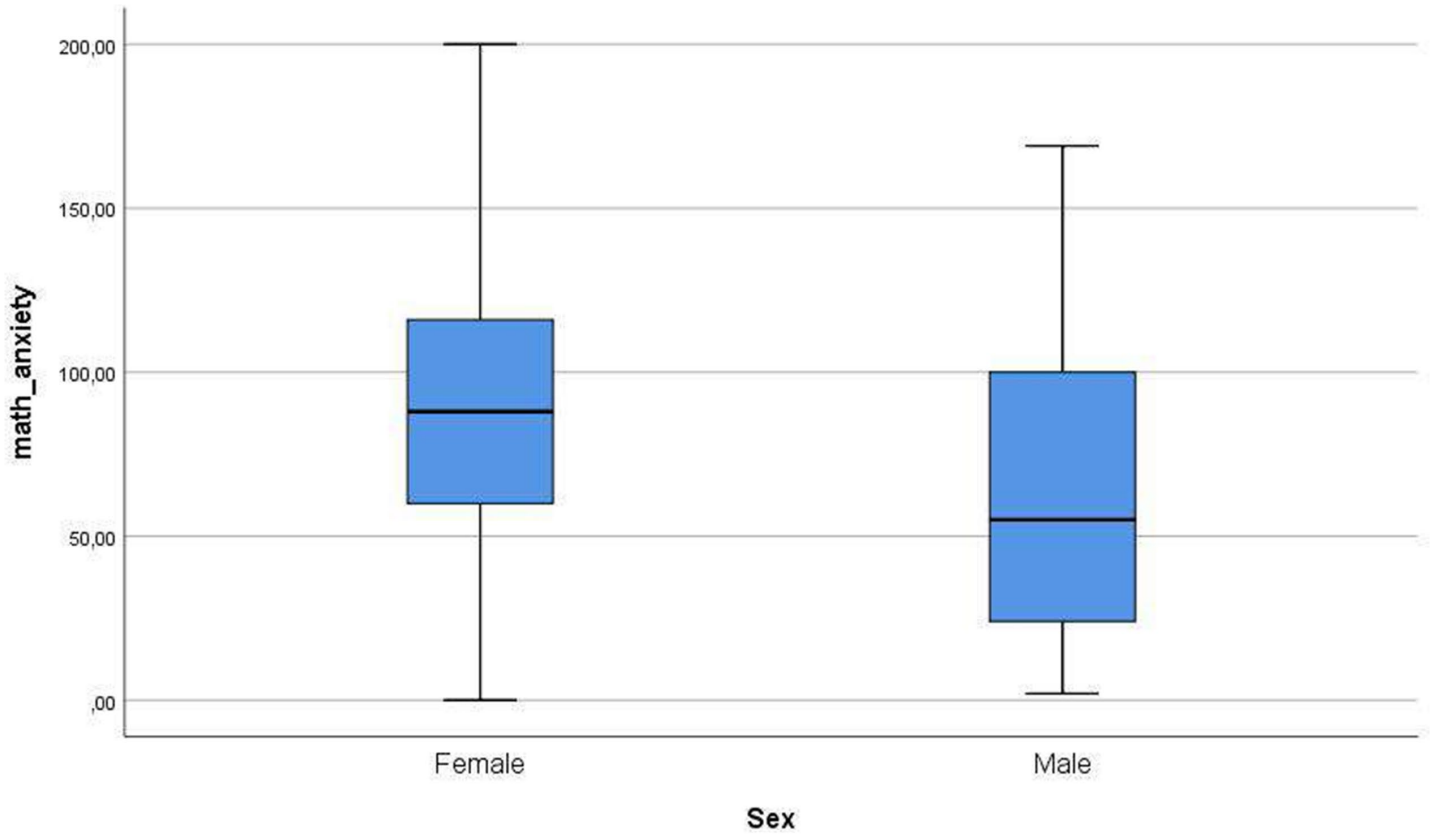
Differences between sexes in levels of mathematical anxiety.

Figures 1–4 below show the differences between sexes in the levels of the variables in this study.

### 3.3. Relationship between mathematics and statistics attitudes and anxiety levels and self-efficacy

The results of the multiple regression models for the dependent variables (mathematics and statistics attitudes) are shown in Tables 2, 3 for the male sample and in Tables 4, 5 for the female sample.

**Table 2:** Multiple linear regression models for the dimensions of the mathematics attitudes (dependent variable) in male.

Independent variables	*B*	CI (95%)		β	SE	*p*
		Lower bound	Upper bound			
Anxiety (*R*^2^ = 0.534)						
Math anxiety in exams	−0.204	−0.340	−0.067	−0.410	0.068	0.004
Anxiety in mathematical learning	−0.129	−0.225	−0.033	−0.367	0.048	0.009
Pleasure (*R*^2^ = 0.193)						
Anxiety in mathematical learning	−0.084	−0.127	−0.042	−0.440	0.021	<0.001
Utility (*R*^2^ = 0.266)						
Anxiety in mathematical learning	−0.122	−0.144	−0.060	−0.516	0.021	<0.001
Motivation (*R*^2^ = 0.118)						
Math anxiety in exams	−0.063	−0.105	−0.200	−0.344	0.021	0.004
Confidence (*R*^2^ = 0.085)						
Everyday mathematical anxiety	−0.040	−0.072	−0.008	−0.292	0.016	0.016

*R*^2^, regression coefficient of determination; B, regression coefficient; CI, confidence interval; β, adjusted coefficient from multiple linear regression analysis; SE, coefficient standard error.

**Table 3:** Multiple linear regression models for the dimensions of the statistics attitudes (dependent variable) in male.

Independent variables	*B*	CI (95%)		β	SE	*p*
		Lower bound	Upper bound			
Safety (*R*^2^ = 0.177)						
Anxiety in mathematical learning	−0.174	−0.267	−0.081	−0.420	0.047	<0.001
Importance (*R*^2^ = 0.082)						
Math anxiety in exams	−0.096	−0.176	−0.016	−0.286	0.040	0.019
Utility (*R*^2^ = 0.051)						
Everyday mathematical anxiety	0.028	−0.024	0.079	0.140	0.026	0.287
Math anxiety in exams	−0.054	−0.113	0.006	−0.236	0.030	0.076
Desire to know (*R*^2^ = 0.084)						
Anxiety regarding mathematical learning	−0.027	−0.063	0.010	−0.180	0.018	0.149
Self-efficacy	0.106	−0.034	0.250	0.186	0.071	0.136

*R*^2^, regression coefficient of determination; B, regression coefficient; CI, confidence interval; β, adjusted coefficient from multiple linear regression analysis; SE, coefficient standard error.

**Table 4:** Multiple linear regression models for the dimensions of the mathematics attitudes (dependent variable) in females.

Independent variables	*B*	CI (95%)		β	SE	*p*
		Lower bound	Upper bound			
Anxiety (*R*^2^ = 0.574)						
Anxiety in mathematical learning	−0.224	−0.277	−0.170	−0.565	0.027	<0.001
Math anxiety in exams	−0.137	−0.223	−0.052	−0.215	0.043	0.002
Self-efficacy	0.236	0.078	0.394	0.134	0.080	0.004
Pleasure (*R*^2^ = 0.263)						
Anxiety in mathematical learning	−0.094	−0.116	−0.073	−0.512	0.011	<0.001
Utility (*R*^2^ = 0.154)						
Anxiety in mathematical learning	−0.089	−0.117	−0.060	−0.392	0.014	<0.001
Motivation (*R*^2^ = 0.149)						
Anxiety in mathematical learning	−0.040	−0.054	−0.027	−0.386	0.007	<0.001
Confidence (*R*^2^ = 0.201)						
Anxiety in mathematical learning	−0.057	−0.079	−0.036	−0.500	0.011	<0.001
Self-efficacy	0.118	0.055	0.181	0.232	0.032	<0.001
Math anxiety in exams	0.039	0.005	0.073	0.212	0.017	0.024

*R*^2^, regression coefficient of determination; B, regression coefficient; CI, confidence interval; β, adjusted coefficient from multiple linear regression analysis; SE, coefficient standard error.

**Table 5:** Multiple linear regression models for the dimensions of the statistics attitudes (dependent variable) in female.

Independent variables	*B*	CI (95%)		β	SE	*p*
		Lower bound	Upper bound			
Safety (*R*^2^ = 0.316)						
Anxiety in mathematical learning	−0.237	−0.285	−0.189	−0.526	0.024	<0.001
Importance (*R*^2^ = 0.111)						
Anxiety in mathematical learning	−0.075	−0.104	−0.046	−0.334	0.015	<0.001
Utility (*R*^2^ = 0.063)						
Math anxiety in exams	−0.068	−0.104	−0.032	−0.252	0.018	<0.001
Desire to know (*R*^2^ = 0.107)						
Anxiety in mathematical learning	−0.054	−0.075	−0.032	−0.327	0.011	<0.001

*R*^2^, regression coefficient of determination; B, regression coefficient; CI, confidence interval; β, adjusted coefficient from multiple linear regression analysis; SE, coefficient standard error.

In summary, for the mathematics attitude dimension of anxiety, 53.4–57.4% of the total variance was predicted; for the liking dimension, 19.3–26.3% of the total variance was predicted; for the utility dimension, 15.4–26.6% was predicted; for the motivation dimension, 11.8–14.9% of the total variance was predicted; and for the confidence dimension, 8.5–20.1% of the total variance was predicted.

For the statistical attitude dimension of security, 17.7–31.6% of the total variance was predicted; for the importance dimension, 8.2–11.1% of the total variance was predicted; for the utility dimension, 5.1–6.3% of the total variance was predicted; and for the will to know dimension, 8.4–10.7% of the total variance was predicted.

### 3.4. Differences in mathematical anxiety before and after a mathematics exam

First, we wanted to test whether the difference between pre- and post-exam mathematical anxiety levels was significant. For this purpose, the Wilcoxon test was applied, showing that, with a significance of 0.028, the difference was sufficient to be considered significant. The difference tended to be positive, showing that post-exam anxiety was lower than pre-exam anxiety.

### 3.5. Academic performance: proposal of an artificial neural network based on self-efficacy, mathematics and statistics attitudes, mathematical anxiety and sociodemographic variables

**Figure 5:**
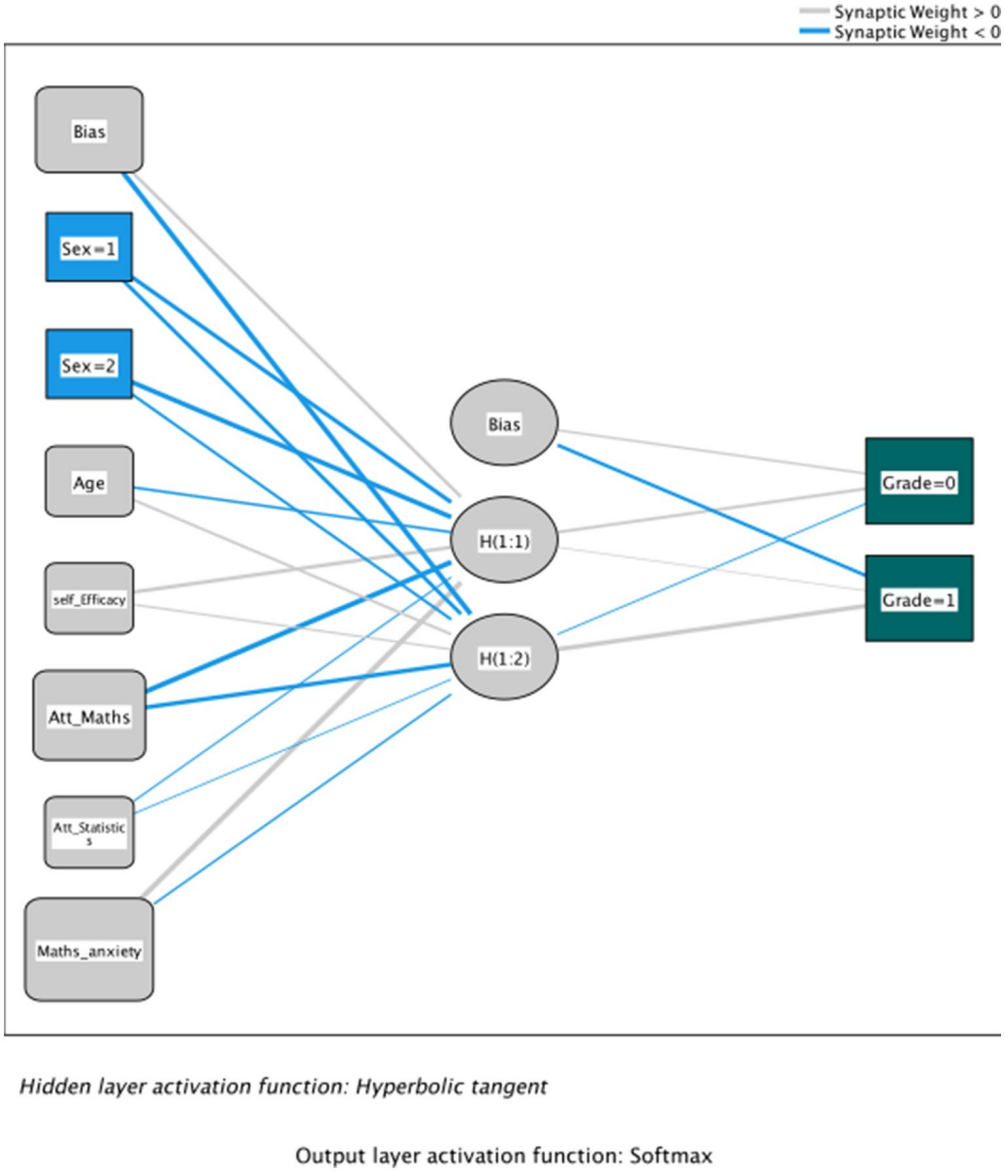
Academic performance: Proposal of an artificial neural network based on self-efficacy, mathematics and statistics attitudes, mathematical anxiety, and sociodemographic variables.

Figure 5 shows the graphical representation of the artificial neural network. Scores below 0 indicate a darker link, whereas scores above 0 indicate a lighter line. In addition, the thickness of the line represents the synaptic weight, the value of which are shown in Table 6, the “Parameter Estimates.”

**Table 6:** Estimated parameters of the artificial neural network.

Predictor		Predicted			
		Hidden layer 1		Output layer	
		H(1:1)	H(1:2)	[Grade = 0]	[Grade = 1]
Input layer	(Bias)	0.305	−0.635		
	[Sex = 1]	−0.362	−0.357		
	[Sex = 2]	−0.602	−0.272		
	Age	−0.176	0.248		
	Self-Efficacy	0.323	0.140		
	Att_Maths	−0.716	−0.433		
	Att_Statistics	−0.127	−0.019		
	Math_anxiety	0.804	−0.143		
Hidden layer 1	(Bias)			0.166	−0.314
	H(1:1)			0.299	0.016
	H(1:2)			−0.059	0.580

Sex = 1, female; Sex = 2, male; Att_Maths, mathematics attitudes; Att_Statistics, statistics attitudes; Grade = 0: Q2, Q3 y Q4 within academic performance; Grade = 1: Q1 or academic excellence.

In the training phase, the cross-entropy error was 110.057 with 27.8% of incorrect forecasts using one consecutive step without error reduction as the stopping rule. In the testing phase, the cross-entropy error was 27.818, and the incorrect forecasts were reduced to 19.3%. Finally, during the reserve phase, the incorrect forecasts were 12.9%. The dependent variable was “grade” (academic excellence), and it was specified that the error calculations were based on a verification sample. Table 6, “Parameter estimates,” shows the synaptic weights in the interactions between the nodes of the different layers.

Table 7, “Classification,” shows how in the training phase, the algorithm was able to predict 72.2% of the cases. Subsequently, when putting the algorithm to the test again and perfecting it, it reached a value of 80%, which was even higher when facing the cases reserved to start the ANN, facing cases that it had not previously analyzed, and reaching a predictive capacity of 87.1%.

**Table 7:** Clasification.

Example	Observed	Predicted		
		Academic performance Q2, Q3 y Q4	Performance of excellence (Q1)	Correct percentage
Training	Academic performance Q2, Q3 y Q4	135	0	100.0%
	Performance of excellence (Q1)	52	0	0.0%
	Overall percentage	100.0%	0.0%	72.2%
Testing	Academic performance Q2, Q3 y Q4	46	0	100.0%
	Performance of excellence (Q1)	11	0	0.0%
	Overall percentage	100.0%	0.0%	80.7%
Holdout	Academic performance Q2, Q3 y Q4	27	0	100.0%
	Performance of excellence	4	0	0.0%
	Overall percentage	100.0%	0.0%	87.1%

Dependent variable: academic excellence.

**Figure 6:**
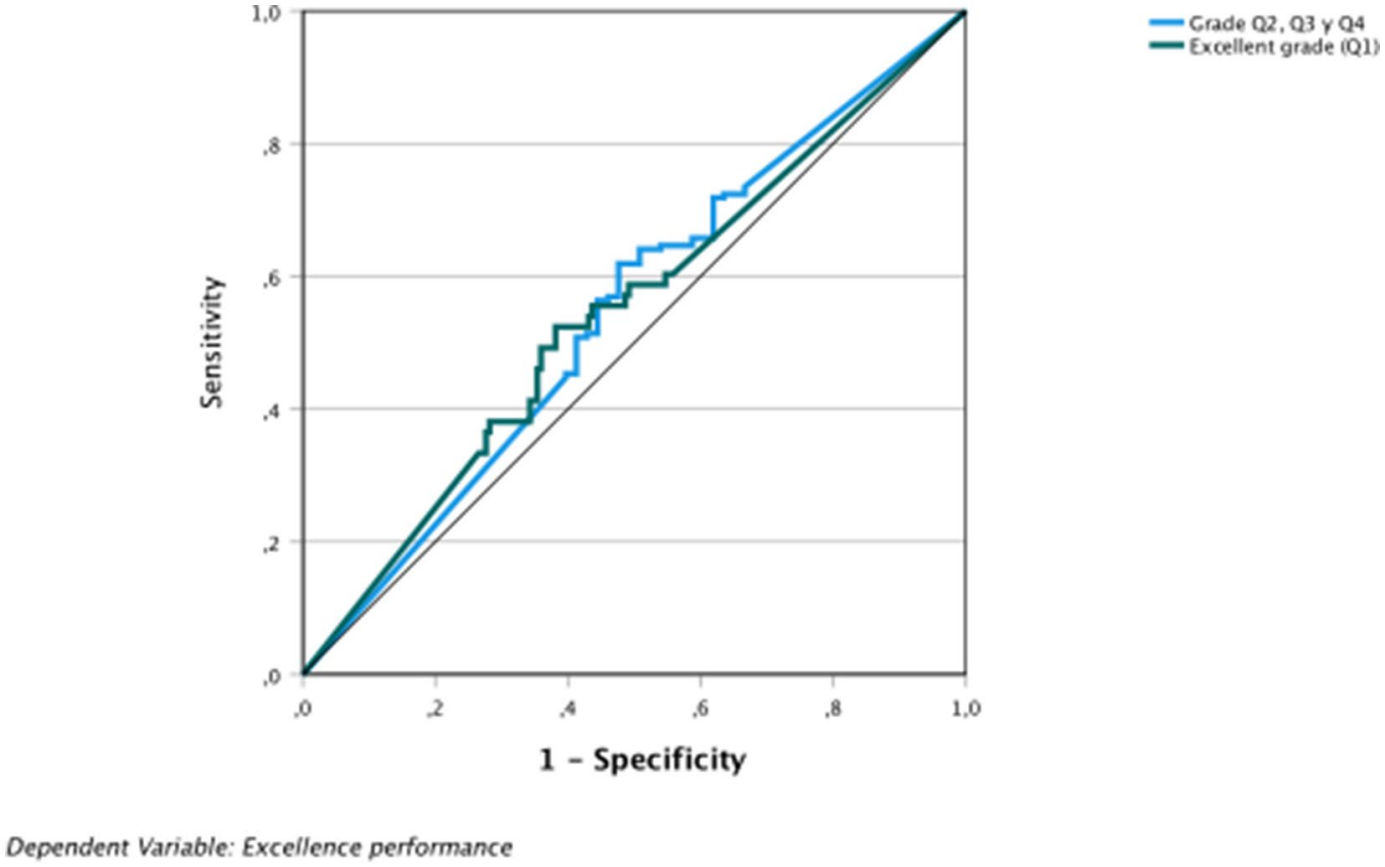
Dependent variable: academic excellence: especificidad.

**Figure 7:**
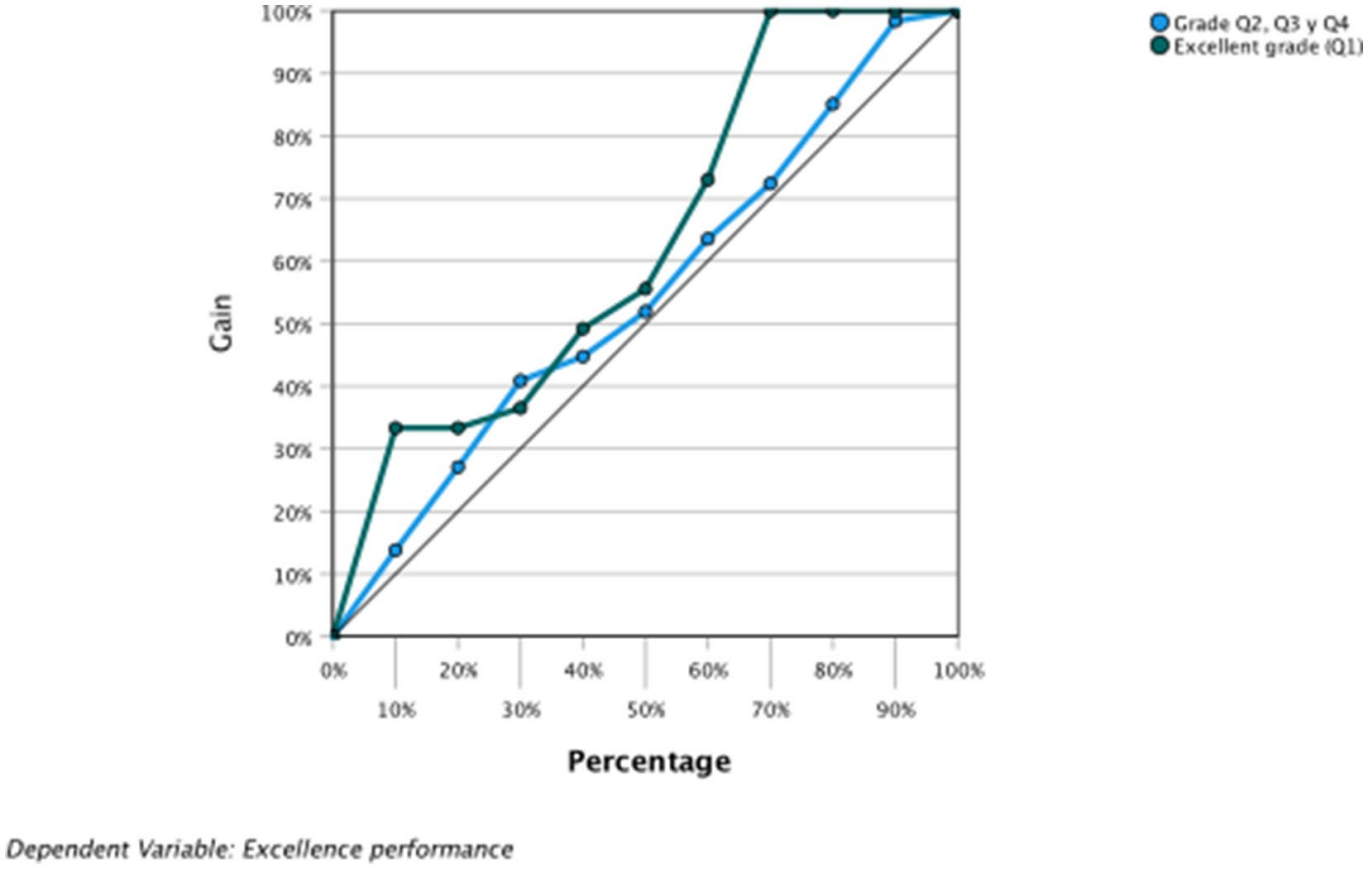
Dependent variable: academic excellence. Porcentages ganancia.

**Figure 8:**
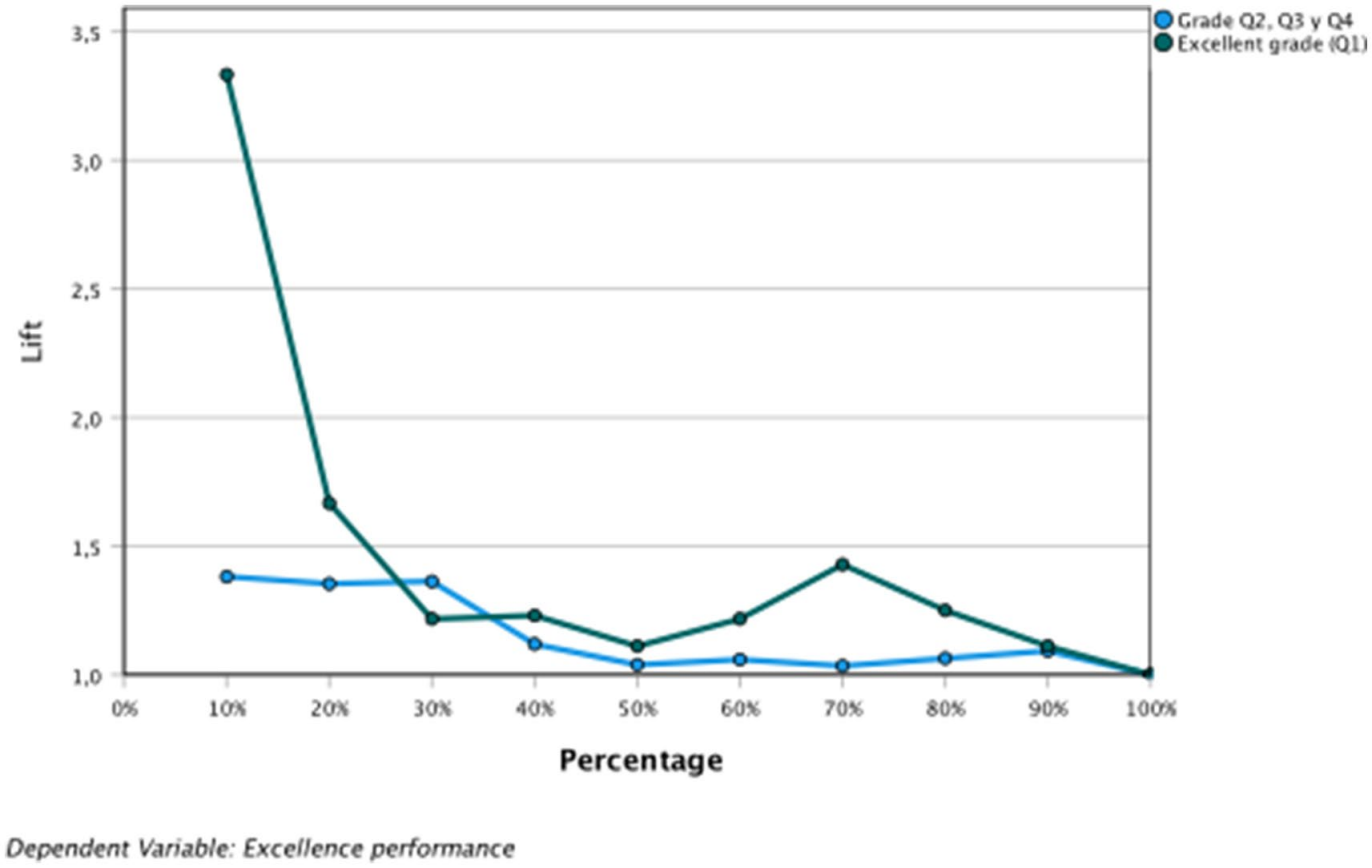
Dependent variable: academic excellence. Porcentages elevation.

The area under the curve is above the diagonal; therefore, the results obtained are not due to chance. In the COR graphs of sensitivity, gain, and elevation, the behavior of the VI was observed; more specifically, VI = 0 and VI = 1 (Figures 6–8 and Table 8).

**Table 8:** Area under the curve.

		Areas
Academic excellence (Grade)	Academic performance Q2, Q3 y Q4	0.545
	Performance of excellence (Q1)	0.544

**Figure 9:**
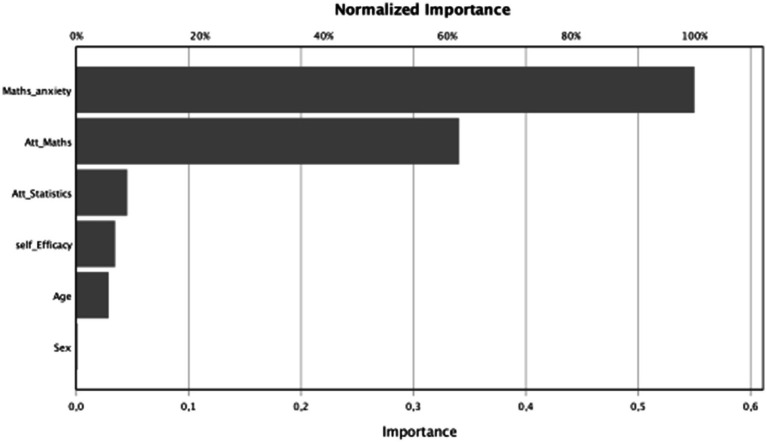
Independent variable: importance.

When analyzing the importance of the independent variables (Table 9 and Figure 9), “mathematics anxiety” was found to be the variable that contributed the most to the predictive capacity of the model, followed by “attitudes toward mathematics” and “attitudes toward statistics.” The sociodemographic variable “sex” barely contributed to predictive capacity.

**Table 9:** Independent variable importance.

	Importance	Normalized importance
Sex	0.001	0.2%
Age	0.029	5.2%
Self-efficacy	0.035	6.3%
Mathematics attitudes	0.341	61.9%
Statistics attitudes	0.045	8.3%
Mathematical anxiety	0.550	100.0%

Dependent variable (VD): Grade.

## 4. Discussion

This study examined the relationship between different aspects of mathematics learning and statistics representing every student. To keep them in mind, we refer to the dimensions above explained of mathematics anxiety, mathematics and statistics attitudes, and finally self-efficacy.

As mentioned at the beginning of this study, some relationships were expected regarding the influence of these variables that are part of the affective-emotional domain on attitudes toward mathematics, in the same way as in the previous study by Xiao and Sun (2021) in which the negative impact of mathematical anxiety on attitudes related to mathematics was evidenced, with students with higher levels of mathematical anxiety being less persistent in the study and with lower academic performance. It was also expected to find, in general terms, the positive relationship between self-efficacy and those attitudes in coherence with the study conducted by Czocher et al. (2020) in which positive correlations were also found between the levels of self-efficacy and the degree of motivation and persistence in studying them.

In this sense, it is important that future teachers are trained to consider not only the cognitive components but also the affective domain so relevant for the teaching/learning process of mathematics as stated in different studies (García-Suárez et al., 2023; Spencer et al., 2023).

### 4.1. Relationship between sex and study variables

Self-efficacy levels are quite similar among males and females in our research. Considering that the maximum level that could be reached was 40, the people asked were themselves thought to be capable of facing a wide range of situations and having tools to solve them properly. Specifically, the average level for the sample was 29.590, which is a high level according to the scale. In addition to that, there is no significant difference between both populations. In the previous study by Abín et al. (2020) significant differences were found according to sex, even considering that the differences were due to the role played by this variable rather than due to the effects of affective and motivational variables. Also, in a recent investigation (Ryan et al., 2022), different levels of mathematical self-efficacy were found as a function of gender. In contrast, in another study, no statistically significant gender differences were found in the self-efficacy variable (Smith et al., 2010). It would be interesting to continue to administer even more specific instruments for the evaluation of this variable in the cultural context in which this study is conducted.

Regarding mathematics attitudes, there was a significant difference between males and females. In fact, males showed a slightly higher level of mathematical attitudes than females. This could be due to several reasons, such as some stereotypes present in the sciences in general, and in mathematics in particular, as stated in Ghazvini and Khajehpour (2011), Rodríguez et al. (2020), and Froehlich et al. (2022). For statistical attitudes, unlike before, there was no significant difference between males and females in this research. This result is congruent with previous studies in which no gender differences in attitudes toward statistics are found (Gil-Flores, 1999; Mondéjar et al., 2007). It seems that it is the previous experience in some way related to statistics, such as having books on this subject at home or having read some of these books, which is most related to the positive attitudes shown toward statistics, especially in terms of liking it (Vilà and Rubio, 2016). This can be explained by an argument regarding utility. In other words, as statistics are, in general, less abstract than other mathematics branches and, as a consequence, more applied to other fields, it is generally more appealing to students.

Finally, there was a significant difference in mathematics anxiety between males and females in our research. In general, the level of state-anxiety is higher in women compared to men (Montero and Morales-Rodríguez, 2021). More specifically, recent research shows higher levels of math anxiety in females compared to males (Rončević Zubković et al., 2021). Other papers also find statistically significant gender differences in levels of math anxiety, with females presenting higher levels (Paechter et al., 2017; Ryan et al., 2022). As previously stated, Ghazvini and Khajehpour (2011), Özcan and Eren Gümüş (2019), Li et al. (2021), Rovan et al. (2022), and Ryan et al. (2022) treat this situation and provide some possible reasons. In this sense, some of the relevant factors that can be highlighted are the training and previous knowledge in this subject by the students, which can be related to the levels of anxiety and stress they show (Vilà and Rubio, 2016).

### 4.2. Relationship between mathematics and statistics attitudes and study variables

For both mathematics and statistics attitudes, there was a similar pattern of influence on mathematics anxiety and self-efficacy in the present study. As expected, in most cases, the influence of mathematical anxiety was mainly negative, which seems plausible and logical if considered Mathematical Anxiety can be interpreted as aversion to mathematics in any form (Li et al., 2021; Ryan et al., 2022). In fact, the dimensions of this kind of anxiety studied here involve many aspects of a student’s life, from daily activities such as buying clothes to taking a mathematics test being as García et al. (2016) indicates higher levels of anxiety when facing an exam or trying to understand a mathematical problem in class before other situations of everyday life in which Mathematics is continuously being applied. The influence of this type of anxiety on students’ learning results in, as previously mentioned, an aversion and a false feeling of incapability in any mathematical knowledge, pygmalion effect, and a less positive mathematical self-concept (Rončević Zubković et al., 2021). Besides, The students with lower levels of mathematical anxiety were found to have higher motivation and performance compared to other motivational profiles with higher levels of anxiety (Xiao and Sun, 2021). The study by Rovan et al. (2022) also indicates that motivational beliefs are a predictor variable of mathematics anxiety in prospective teachers. In another recent research, García-Suárez et al. (2023) show the relationship between the mathematical anxiety construct and the greater presence of negative attitudes and their consequences for the learning of this subject. It can also be noted that, as found by Paechter et al. (2017), it can be expected that levels of anxiety toward mathematics can be expected to correlate positively with levels of statistical anxiety.

However, the opposite was observed in the self-efficacy dimension. In all cases where this dimension is significant, the effect produced can be categorized as positive. A positive evaluation of this dimension may be crucial in terms of the capability to face and solve problems (Chen et al., 2023; Poçan et al., 2023; SalehAlabdulaziz, 2023; Samuelsson, 2023). It is also a predictor variable that has been considered relevant for motivation and persistence for the so-called STEAM careers and hence the need to contribute to its improvement from different educational levels (Czocher et al., 2020). These data obtained in our study are congruent with other research in which levels in the variable self-efficacy were found to be related to students’ levels of math anxiety and academic motivation (Özcan and Eren Gümüş, 2019). Recently, García-Suárez et al. (2023) found inverse correlations between the variable’s math anxiety and perceived degree of self-confidence.

### 4.3. Differences in mathematical anxiety before and after a mathematics exam

The results showed that levels of mathematical anxiety decreased after the completion of a mathematics test. This can be understood as a sensation of freedom and relief that appeared after the exam was completed. However, this sensation is supposed to be transient as worries appear again when students face mathematics lessons. The academic setting is one of those that can generate daily stress in young people (Elsalem et al., 2020; Morales-Rodríguez, 2021) and, like the current study, in the work of García et al. (2016), it is mentioned that the university students participating in their research show less anxiety toward mathematics in their daily lives compared to when they try to understand mathematical problems, or when they sit for evaluations or exams corresponding to the mathematics subjects they take in their academic training.

### 4.4. Relationship between academic performance and mathematical anxiety and mathematical and statistics attitudes: proposal of an artificial neural network based on self-efficacy, mathematics and statistics attitudes, mathematical anxiety, and sociodemographic variables

The results showed that mathematical anxiety had a crucial paper in the classification in terms of academic performance. In addition to that, mathematical attitudes had also a significant role in the classification. On the other hand, statistical attitudes did not have such importance, but a lesser important impact. The relations, as it is stated in the introduction, are then accepted by the results obtained as evidenced in studies such as those conducted by Xiao and Sun (2021). Geisler et al. (2023) also highlight the existence of relationships between attitudes toward mathematics and the academic achievement variable. Orbach et al. (2019) also find inverse correlations between components of the math anxiety and academic performance variables. In the same line, another research found that anxiety levels and motivation in that order were predictor variables of the performance variable (Suren and Kandemir, 2020). Spencer et al. (2023) found that self-efficacy was also a predictor variable of overall course grade. In our study, the results obtained have been confirmed using the fundamentals of a classical methodology based on regression analysis and, especially, with the innovative design of artificial neural networks, which have proven to be very useful in the field of educational psychology (Hwang et al., 2020; Martínez-Ramón et al., 2021). This confirmation with this type of analysis based on artificial intelligence to the educational field, was much needed in this field of knowledge since there were still results that did not find effects of self-efficacy improvement on academic performance (Gamlem et al., 2019), or that consider the existence of contradictory results regarding the relationships between attitudes toward mathematics such as interest and academic performance (Rach and Heinze, 2017).

### 4.5. Implications for education

Several studies have demonstrated the implications of mathematics anxiety in the learning process. The present research is also one of these, since mathematics and statistics attitudes are crucial to the learning process mentioned above. In this sense, we could emphasize the implications of this research for the advancement of educational psychology by contributing in some way with a new analysis based on artificial intelligence models to further clarify the relationships between constructs related to the affective-emotional dimension of attitudes toward mathematics such as mathematical anxiety and self-efficacy with student motivation and academic achievement. To date, regression models had been applied using structural equations in which there were still inconclusive or contradictory data. Thus, we are trying to contribute to generate new knowledge considering another key aspect to highlight the implications of this study in the field of artificial intelligence and artificial neural networks. The development of the neural network designed based on self-efficacy, mathematics and statistics attitudes, mathematical anxiety, and sociodemographic variables is considered useful in the field of educational psychology, as there are many advantages that the advances in artificial intelligence can bring to the analysis of the relationships between the constructs evaluated here and educational decision-making (Colchester et al., 2016; Guo et al., 2021; Martínez-Ramón et al., 2023). The levels of math anxiety, self-efficacy and sociodemographic variables of the study contributed to the predictive capacity of the artificial neural network of the variable academic performance in this subject.

Mathematical and statistical skills, such as information arrangement and interpretation, are key points in modern society. As we are exposed to uncountable information sources, the process of learning mathematics becomes a recurrent issue when reforms of the educational system are discussed. Precisely in Spain, these aspects are being debated in Early Childhood Education in view of the changes in the curriculum due to new legislative provisions such as the Royal Decree 95/2022, of February 1 (Royal Decree, 2022), which establishes the organization and minimum teachings of Early Childhood Education. Likewise, the LOMLOE (2020) (Organic Law 3/2020, of December 29, which modifies Organic Law 2/2006, of May 3, on Education) places special emphasis on affective-emotional education and the prevention of gender stereotypes within the Mathematics curriculum. Therefore, teacher training in this area is essential to contemplate the design of learning situations that work on emotional competence in related areas and STEAM careers.

Dimensions such as motivation or confidence should be considered in the design of experiences, activities, and didactical units. Thus, the inclusion of new technologies, such as smartphones, personal computers, and other devices, might be interesting, as presented by Plenty and Heubeck (2013), Turel and Sanal (2018), and Watt et al. (2019). In this sense, it should be noted that the use of Information and Communication Technologies with the design of e-learning courses, mobile applications to clarify doubts and difficulties in the teaching/learning process of mathematics, sharing daily news and stories of daily life that demonstrate its usefulness and that it is not inert knowledge, with active and interactive methodologies with the use of images (movies, videos, sharing presentations, etc.) and not only words, could contribute to promote the development and acquisition of this affective or emotional dimension in its positive aspect with the improvement of self-efficacy and in its negative aspect with the improvement of self-efficacy and in its positive aspect with the improvement of self-efficacy, videos, sharing presentations, etc. For this, in the didactic planning it would be necessary to train effective coping strategies to reduce anxiety problems in mathematics exams and to improve self-perceived competences that could help to reduce school difficulties or failure. In addition to that, Ren et al. (2016) described a curious experience. This study examined, in a sample of 94 students in a mathematics course at a Turkish University, how the use of an e-book influenced academic performance, motivation, and mathematical anxiety in a group of students compared to another group that used a printed book. In this sense, in a future study, we could also consider how the use of digital materials and even social networks influences university students. This type of resource would be especially motivating for secondary school students. There are already previous studies that show that having books and reading them about statistics can help to improve attitudes toward mathematics and statistics by generating greater interest and enjoyment (Vilà and Rubio, 2016).

Finally, Orbach et al. (2019) focused on the motivational profile of students. This study explored the relationship between this motivational profile and student aspirations. Data from the *Study of Transitions and Education Pathways* (*STEPS*; Watt et al., 2019) can be consulted. In this sense, this type of assessment related to the affective or emotional dimension of attitudes toward mathematics and self-efficacy is necessary especially in STEAM degrees where, as found by Czocher et al. (2020), academic self-efficacy is one of the most important variables for dropout and contributing to motivation in such degrees. This aspect can be considered especially relevant since achievements in the field related to the teaching of Mathematics are also fundamental for the achievement of the Sustainable Development Goals (2023) given its applications so relevant to everyday life in addition to other aspects related to the gender perspective and for the achievement of goal number 5 of the United Nations 2030 Agenda for Sustainable Development (gender equality and empowerment of women and girls). This would allow us to design interventions and teaching planning considering the effect of the gender variable and the already known Vygotskian Zone of Proximal Development also considering that, as indicated in the study by Spencer et al. (2023) an overconfidence with too easy tasks could also have a negative impact on academic performance. It also allows us to reflect on the importance of the tasks that teachers ask of their students for the acquisition or development of competencies and their feedback in the design of learning situations. Self-regulated learning, self-perceived competencies and metacognition need to be enhanced, consistent with what is put forward by Gamlem et al. (2019) and Özcan and Eren Gümüş (2019). The development of adaptive beliefs about this material should also be encouraged from the initial training of future teachers (Rovan et al., 2022), providing them with the necessary training to avoid gender bias in the assessment of attitudes toward mathematics (León-Mantero et al., 2020). This will be very useful to prevent this discriminatory typology from Early Childhood Education and for the construction of the person and the integral development of the personality, considering in this case the affective or emotional dimension of attitudes toward mathematics in coherence with the LOMLOE. We believe that these aspects can contribute to the necessary improvement of educational inclusion and attention to motivational diversity without leaving emotional competencies in the Mathematics and Statistics curriculum in the background. For the improvement of motivation in the design of learning situations required by the recent laws in Spain, it is considered very useful the keys that are raised in articles such as Valle et al. (2006). Therefore, the information provided by this type of study is useful for vocational guidance of students from secondary education and encourages women to enroll more in degrees such as Mathematics considering these aspects and the importance of improving training and experiences with mathematics and statistics at these educational levels.

### 4.6. Limitations and future research directions

This research could lead to other similar research, as the influence of anxiety on the learning process is an interesting topic. In addition, mathematics has always been a special subject; it is considered a difficult and incomprehensible branch of knowledge, and experts have tried to make it accessible to the majority of the population. For this purpose, the effects of anxiety on the learning process could be studied further and some solutions could be proposed. In addition, activities to improve mathematics and statistics attitudes could be proposed and tested, as these attitudes are important in terms of the predisposition to learn and comprehend. Further studies could include the insertion of these studies and predictions into recently proposed skill-based learning and how the levels of attitudes and anxiety evolve under this type of learning.

One limitation of this study was the sample size in one of its groups, such as the need to continue expanding the sample of secondary education students in more centers, even analyzing differences between centers in rural and urban contexts so that the information can be generalized to other cultures and language registers since the current sample was made up of Spanish-speaking students. On another note, the area under the curve obtained in the ANN also leads to be cautious in its analysis although it has a predictive capacity above chance.

In future studies, it would also be interesting to analyze the affective domain such as anxiety toward mathematics not only in future teachers and students, but also in the other element of the educational situation such as teachers who already teach mathematics. In this sense, in the case of secondary education teachers of mathematics, it has been found that the main situations generating emotional feelings in Mexican teachers were the attitudes of commitment, motivation and the academic performance of their students. Emotions such as pride, gratitude, appreciation, and happiness appeared in the face of student learning, and academic achievement. Non-commitment and low academic motivation generated in teachers’ emotions such as anger, disappointment or reproach (Martínez-Sierra et al., 2022). It would also be interesting to test the effect that the variables cognitive flexibility and creativity, which are predictive of positive attitudes toward mathematics, may have on attitudes toward mathematics in future teachers (De la Peña et al., 2021). In this way, we would not be leaving aside the cognitive domain of mathematics so that, as proposed by De la Peña et al. (2021), we could try to introduce elements of innovation for the improvement of creativity and divergent thinking with implications for the planning of the practical teaching of mathematics that could result in more positive attitudes. Evaluations could also be applied with other more recent instruments even more specifically adapted to the Spanish context for the evaluation of attitudes toward mathematics, such as considering the importance of each item in the factorial estimation by means of a structural equation analysis (León-Mantero et al., 2020) or by means of one of the methods recently considered among the most effective for analyzing the psychometric properties of an instrument, such as the Rash model (Morales-Rodríguez et al., 2021). It would also be interesting to further evaluate cross-cultural variations for comparing different regions.

## Data availability statement

The raw data supporting the conclusions of this article will be made available by the authors, without undue reservation.

## Ethics statement

The studies involving human participants were reviewed and approved by The Ethics Committee of the University of Granada (Granada, Spain, 3376/CEIH/2023). Written informed consent to participate in this study was provided by the participants’ legal guardian/next of kin.

## Author contributions

FM-R led, conceived and designed the study, contributed to the bibliographic review, recruited the participants, and contributed to the manuscript writing and data analysis. JH and JM-R contributed to the manuscript writing, and data analysis. JR-G recruited the secondary school students and contributed to the manuscript writing. All authors revised the manuscript critically and approved the final version of the manuscript.

## Conflict of interest

The authors declare that the research was conducted in the absence of any commercial or financial relationships that could be construed as a potential conflict of interest.

The reviewer CR declared a shared affiliation with the authors JM-R to the handling editor at the time of review.

## Publisher’s note

All claims expressed in this article are solely those of the authors and do not necessarily represent those of their affiliated organizations, or those of the publisher, the editors and the reviewers. Any product that may be evaluated in this article, or claim that may be made by its manufacturer, is not guaranteed or endorsed by the publisher.

## References

[ref1] Abín A. Núñez J. C. Rodríguez C. Cueli M. García T. Rosário P. (2020). Predicting mathematics achievement in secondary education: the role of cognitive, motivational, and emotional variables. Front. Psychol. 11:876. doi: 10.3389/fpsyg.2020.00876, PMID: 32528351 PMC7264990

[ref2] Auzmendi E. (1992). Las actitudes hacia la matemática-estadística en las enseñanzas medias y universitarias. Bilbao: Mensajero.

[ref3] Ávila-Toscano J. H. Rojas-Sandoval Y. Tovar-Ortega T. (2020). Perfil del dominio afectivo en futuros maestros de matemáticas. Revis. Psicol. Educ. 15, 225–236. doi: 10.23923/rpye2020.02.197

[ref4] Baessler J. Schwarzer R. (1996). Evaluación de la autoeficacia; Adaptación española de la escala de autoeficacia general [assessing self-efficacy: Spanish adaption of the general self-efficacy scale]. Ansiedad Estrés 2, 1–8.

[ref9001] Baloğlu M. Zelhart P. F. (2007). Psychometric Properties of the Revised Mathematics Anxiety Rating Scale. Psychol. Rec. 57, 593–611. doi: 10.1007/BF03395597

[ref6] Chen M. F. Chen Y. C. Zuo P. Y. Hou H. (2023). Design and evaluation of a remote synchronous gamified mathematics teaching activity that integrates multi-representational scaffolding and a mind tool for gamified learning. Educ. Inf. Technol. 1-27. doi: 10.1007/s10639-023-11708-6 PMC10037408 37361748

[ref7] Colchester K. Hagras H. Alghazzawi D. Aldabbagh G. (2016). A survey of artificial intelligence techniques employed for adaptive educational systems within E-learning platforms. J. Artif. Intell. Soft Comput. Res. 7, 47–64. doi: 10.1515/jaiscr-2017-0004

[ref8] Cruise R. Cash R. Bolton D. (1985). Development and validation of an instrument to measure statistical anxiety, In Proceedings of the American Statistical Association, section on statistical education, Las Vegas, NV

[ref9] Czocher J. A. Melhuish K. Kandasamy S. S. (2020). Building mathematics self-efficacy of STEM undergraduates through mathematical modelling. Int. J. Math. Educ. Sci. Technol. 51, 807–834. doi: 10.1080/0020739X.2019.1634223

[ref10] Darias Morales E. J. (2000). Attitudes toward statistics [Escala de actitudes hacia la estadística]. Psicothema 12, 175–178.

[ref11] De la Peña C. Fernádez-Cézar R. Solano-Pinto N. (2021). Attitude toward mathematics of future teachers: how important are creativity and cognitive flexibility? Front. Psychol. 12:713941. doi: 10.3389/fpsyg.2021.713941, PMID: 34305767 PMC8302141

[ref12] Elsalem L. al-Azzam N. Jum'ah A. A. Obeidat N. Sindiani A. M. Kheirallah K. A. (2020). Stress and behavioral changes with remote E-exams during the COVID-19 pandemic: a cross-sectional study among undergraduates of medical sciences. Ann. Med. Surg. 60, 271–279. doi: 10.1016/j.amsu.2020.10.058, PMID: 33163179 PMC7604013

[ref13] Emmioğlu E. Çapa-Aydin Y. (2012). Attitudes and achievement in statistics: a meta-analysis study. Stat. Educ. Res. J. 11, 95–102. doi: 10.52041/serj.v11i2.332

[ref14] Espada J. P. Gonzálvez M. T. Orgilés M. Carballo J. L. Piqueras J. A. (2012). Validación de la escala de autoeficacia general con adolescentes españoles [validation of the general self-efficacy scale in Spanish teenagers]. Electron. J. Res. Educ. Psychol. 10, 355–370. doi: 10.25115/ejrep.v10i26.1504

[ref15] Fennema E. Sherman J. A. (1976). Fennema-Sherman mathematics attitude scales: instruments designed to measure attitudes toward the learning of mathematics by females and males. J. Res. Math. Educ. 7, 324–326.

[ref16] Feregrino G. R. López J. A. J. Gómez O. L. F. (2021). Importancia del estudio de las actitudes para el aprendizaje de las matemáticas. RD-ICUAP 7, 148–157.

[ref17] Flores W. O. Auzmendi E. (2015). Análisis de la estructura factorial de una escala de actitud hacia las matemáticas [analysis of the factorial structure of an attitude towards mathematics scale]. Aula Encuentro 17, 45–77. doi: 10.6018/rie.359991

[ref18] Froehlich L. Tsukamoto S. Morinaga Y. Sakata K. Uchida Y. Keller M. M.. (2022). Gender stereotypes and expected backlash for female STEM students in Germany and Japan. Front. Educ. 6:793486. doi: 10.3389/feduc.2021.793486

[ref19] Galende N. Arrivillaga A. R. Madariaga J. M. (2020). Attitudes towards mathematics in secondary school students. Personal and family factors (las actitudes hacia las matemáticas del alumnado de secundaria. Factores personales yfamiliares). Cult. Educ. 32, 529–555. doi: 10.1080/11356405.2020.1785156

[ref20] Gamlem S. Kvinge L. M. Smith K. Engelsen K. S. (2019). Developing teachers’ responsive pedagogy in mathematics, does it lead to short-term effects on student learning? Cogent Educ. 6:1676568. doi: 10.1080/2331186X.2019.1676568

[ref21] García E. Schnell J. Ramos J. (2016). Anxiety towards mathematics on undergraduates in a nautical school (an empirical study in Port Veracruz). Matemath. Educ. 11, 2418–2429.

[ref22] Garcia-Manrubia B. Méndez I. García-Montalbán J. (2022). Evolución de las actitudes hacia las matemáticas en estudiantes universitarios [evolution of attitudes towards mathematics in university students]. Eur. J. Develop. Educa. Psychop 10, 1–10. doi: 10.32457/ejpad.v10i1.2069

[ref23] García-Suárez J. Guzmán-Martínez M. Monje-Parrilla F. J. (2023). Estudio descriptivo de la ansiedad matemática en estudiantes mexicanos de ingeniería. IE Revist. Investig. Educ. REDIECH 14:e1619. doi: 10.33010/ie_rie_rediech.v14i0.1619

[ref24] Geisler S. Rach S. Rolka K. (2023). The relation between attitudes towards mathematics and dropout from university mathematics—the mediating role of satisfaction and achievement. Educ. Stud. Math. 112, 359–381. doi: 10.1007/s10649-022-10198-6

[ref25] Ghazvini S. D. Khajehpour M. (2011). Gender differences in factors affecting academic performance of high school students. Procedia. Soc. Behav. Sci. 15, 1040–1045. doi: 10.1016/j.sbspro.2011.03.236

[ref26] Gil-Flores J. (1999). Actitudes hacia la Estadística. Incidencia de las variables sexo y formación previa. Rev. Espanola de Pedagog. 214, 567–590.

[ref27] Guo J. Bai L. Yu Z. Zhao Z. Wan B. (2021). An AI-ApplicationOriented in-class teaching evaluation model by using statistical modeling and ensemble learning. Sensors 21, 1–28. doi: 10.3390/s21010241, PMID: 33401482 PMC7796427

[ref28] Hossein-Mohand H. Hossein-Mohand H. (2023). Influence of motivation on the perception of mathematics by secondary school students. Front. Psychol. 13:1111600. doi: 10.3389/fpsyg.2022.1111600 36743609 PMC9893926

[ref29] Hwang G. J. Xie H. Wah B. W. Gašević D. (2020). Vision, challenges, roles and research issues of artificial intelligence in education. Comput. Educ. Artif. Intel. 1:100001. doi: 10.1016/j.caeai.2020.100001

[ref30] Laux L. Glanzmann P. Schaffner P. Spielberger C. (1981). Das State-Trait-Angstinventar. Theoretische Grundlagen und Handanweisung Weinheim: Beltz Test GmbH.

[ref31] León-Mantero C. Casas-Rosal J. C. Pedrosa J. C. Maz-Machado A. (2020). Measuring attitude towards mathematics using Likert scale surveys: the weighted average. PLoS One 15:e0239626. doi: 10.1371/journal.pone.0239626 33002073 PMC7529215

[ref32] Li Q. Cho H. Cosso J. Maeda Y. (2021). Relations between students’ mathematics anxiety and motivation to learn mathematics: a meta-analysis. Educ. Psychol. Rev. 33, 1017–1049. doi: 10.1007/s10648-020-09589-z

[ref33] Lim S. Chapman E. (2015). Effects of using history as a tool to teach mathematics on students’ attitudes, anxiety, motivation and achievement in grade 11 classrooms. Educ. Stud. Math. 90, 189–212. doi: 10.1007/s10649-015-9620-4

[ref34] LOMLOE (2020). Organic law 3/2020, of December 29, amending organic law 2/2006, of may 3, 2006, on education (s/f). Available at: https://www.boe.es/eli/es/lo/2020/12/29/3 (Accessed March 28, 2023)

[ref35] Macher D. Paechter M. Papousek I. Ruggeri K. (2012). Statistics anxiety, trait anxiety, learning behavior, and performance. Eur. J. Psychol. Educ. 27, 483–498. doi: 10.1007/s10212-011-0090-5

[ref36] Martínez-Ramón J. P. Morales-Rodríguez F. M. Pérez-López S. (2021). Burnout, resilience, and COVID-19 among teachers: predictive capacity of an artificial neural network. Appl. Sci. 11:8206. doi: 10.3390/app11178206

[ref9002] Martínez-Ramón J. P. Morales-Rodríguez F. M. Pérez-López S. Méndez Mateo I Ruiz-Esteban C. (2023). Predicción de la resiliencia docente mediante redes neuronales artificiales: influencia del burnout y del estrés por COVID-19. Anales de Psicología / Annals of Psychology, 39:100–111. doi: 10.6018/analesps.515611

[ref37] Martínez-Sierra G. Arellano-García Y. Hernández-Moreno A. (2022). Which situations trigger emotions of secondary school mathematics teachers? Int. J. Sci. Math. Educ. 20, 575–595. doi: 10.1007/s10763-021-10158-1

[ref38] Mondéjar J. Vargas M. Mondéjar J. A. (2007). Impacto del uso del e-learning en las actitudes hacia la Estadística. Revist. Latinoamericana Tecnol. RELATEC Educ. 6, 31–47.

[ref39] Montero E. S. Morales-Rodríguez F. M. (2021). Evaluation of anxiety, suicidal risk, daily stress, empathy, perceived emotional intelligence, and coping strategies in a sample of Spanish undergraduates. Int. J. Environ. Res. Public Health 18:1418. doi: 10.3390/ijerph18041418, PMID: 33546459 PMC7913637

[ref40] Morales-Rodríguez F. M. (2021). Fear, stress, resilience, and coping strategies during COVID-19 in Spanish university students. Sustainability 13:5824. doi: 10.3390/su13115824

[ref41] Morales-Rodríguez F. M. Martí-Vilar M. Narváez-Peláez M. A. Giménez-Lozano J. M. Martínez-Ramón J. P. Caracuel A. (2021). Psychometric properties of the affective dimension of the generic macro-competence assessment scale: analysis using Rasch model. Sustainability 13:6904. doi: 10.3390/su13126904

[ref42] O’Callaghan J. Essau C. Ederer E. Bokszczanin A. Sasagawa S. (2009). “Academic and general procrastination: a cross-cultural comparison” in Teaching psychology around the world. eds. McCarthy S. Karandashev V. Stevens M. Thatcher A. Jaafar J. Moore K.., vol. 2 (Newcastle upon Tyne:Cambridge Scholars Publishing), 341–360.

[ref43] Orbach L. Herzog M. Fritz A. (2019). Relation of state- and trait-math anxiety to intelligence, math achievement and learning motivation. J. Numer. Cogn. 5, 371–399. doi: 10.5964/jnc.v5i3.204

[ref44] Özcan Z. Ç. Eren Gümüş A. (2019). A modeling study to explain mathematical problem-solving performance through metacognition, self-efficacy, motivation, and anxiety. Aust. J. Educ. 63, 116–134. doi: 10.1177/0004944119840073

[ref45] Paechter M. Macher D. Martskvishvili K. Wimmer S. Papousek I. (2017). Mathematics anxiety and statistics anxiety. Shared but also unshared components and antagonistic contributions to performance in statistics. Front. Psychol. 8:1196. doi: 10.3389/fpsyg.2017.01196, PMID: 28790938 PMC5522867

[ref46] Papousek I. Ruggeri K. Macher D. Paechter M. Heene M. Schulter G.. (2012). Psychometric evaluation and experimental validation of the statistics anxiety rating scale. J. Pers. Assess. 94, 82–91. doi: 10.1080/00223891.2011.627959, PMID: 22176269

[ref47] Plenty S. Heubeck B. G. (2013). A multidimensional analysis of changes in mathematics motivation and engagement during high school. Educ. Psychol. 33, 14–30. doi: 10.1080/01443410.2012.740199

[ref48] Poçan S. Altay B. Yaşaroğlu C. (2023). The effects of Mobile technology on learning performance and motivation in mathematics education. Educ. Inf. Technol. 28, 683–712. doi: 10.1007/s10639-022-11166-6, PMID: 35814805 PMC9253263

[ref49] Rach S. Heinze A. (2017). The transition from school to university in mathematics: which infuence do school-related variables have? Int. J. Sci. Math. 15, 1343–1363. doi: 10.1007/s10763-016-9744-8

[ref50] Ren L. Green J. L. Smith W. M. (2016). Using the Fennema-Sherman mathematics attitude scales with lower-primary teachers. Math. Ed. Res. J. 28, 303–326. doi: 10.1007/s13394-016-0168-0

[ref51] Rodríguez S. Regueiro B. Piñeiro I. Estévez I. Valle A. (2020). Gender differences in mathematics motivation: differential effects on performance in primary education. Front. Psychol. 10:3050. doi: 10.3389/fpsyg.2019.03050, PMID: 32063870 PMC7000542

[ref52] Rončević Zubković B. Pahljina-Reinić R. Kolić-Vehovec S. (2021). Age and gender differences in mathematics learning during school transition. Int. J. School Educ. Psychol. 11, 20–33. doi: 10.1080/21683603.2021.1934206

[ref53] Rosenbluth A. Cruzat-Mandich C. Ugarte M. L. (2016). Methodology to validate a competencies assessment tool for psychology students. Univ. Psychol. 15, 303–314. doi: 10.11144/Javeriana.upsy15-1.ppmp

[ref54] Rovan D. Trupčević G. Glasnović Gracin D. (2022). Motivacija za učenje matematike kod budućih učitelja razredne nastave. Društvena Istraživanja 31, 113–133. doi: 10.5559/di.31.1.06

[ref55] Royal Decree (2022). Royal Decree 95/2022, of February 1, 2022, which establishes the organization and minimum teachings of early childhood education. Official State Gazette, No. 28, February 1. Available at: https://www.boe.es/eli/es/rd/2022/02/01/95/com

[ref56] Ryan V. Fitzmaurice O. O’Donoghue J. (2022). Student interest and engagement in mathematics after the first year of secondary education. Eur. J. Sci. Math. Educ. 10, 436–454. doi: 10.30935/scimath/12180

[ref57] SalehAlabdulaziz M. (2023). Escape rooms technology as a way of teaching mathematics to secondary school students. Educ. Inf. Technol. 29, 1–26. doi: 10.1007/s10639-023-11729-1 PMC10052216 37361796

[ref58] Samuelsson J. (2023). Developing students' relationships with mathematics. Educ. Action Res. 31, 180–194. doi: 10.1080/09650792.2021.1899012

[ref59] Sánchez J. Segovia I. Miñán A. (2022). Ansiedad matemática, rendimiento y formación de acceso en futuros maestros. PNA 16, 115–140. doi: 10.30827/pna.v16i2.21703

[ref60] Smith L. Sinclair K. E. Chapman E. S. (2010). Students’ goals, self-efficacy, self-handicapping, and negative affective responses: an Australian senior school student study. Contemp. Educ. Psychol. 27, 471–485. doi: 10.1006/ceps.2001.1105

[ref61] Spencer D. Griffith E. Briska K. Post J. Willis C. (2023). The role of non-cognitive factors in the introductory statistics classroom. Stat. Educ. Res. J. 22, 1–16. doi: 10.52041/serj.v22i1.77

[ref62] Suren N. Kandemir M. A. (2020). The effects of mathematics anxiety and motivation on students' mathematics achievement. Int. J. Educ. Math. Sci. Technol. 8, 190–218. doi: 10.46328/ijemst.v8i3.926

[ref63] Sustainable Development Goals (2023). Sustainable Development Goals of the United Nations 2030 Agenda. Available at: https://www.un.org/sustainabledevelopment/es/sustainable-development-goals/

[ref64] Turel Y. K. Sanal S. O. (2018). The effects of an ARCS based e-book on student's achievement, motivation and anxiety. Comput. Educ. 127, 130–140. doi: 10.1016/j.compedu.2018.08.006

[ref65] Valle A. Cabanach R. G. Susana Rodríguez S. Núñez J. C. González-Pienda J. A. (2006). Metas académicas, estrategias cognitivas y estrategias de autorregulación del estudio. Psicothema 18, 165–170. doi: 10.18774/448x.2009.6.60 PMID: 17296027

[ref66] Vilà R. Rubio M. J. (2016). Actitudes hacia la Estadística en el alumnado del grado de Pedagogía de la Universidad de Barcelona. Revist. Docencia Univers. REDU 14, 131–149. doi: 10.4995/redu.2016.5766

[ref67] Watt H. M. G. Bucich M. Dacosta L. (2019). Adolescents' motivational profiles in mathematics and science: associations with achievement striving, career aspirations and psychological 2ellbeing. Front. Psychol. 10, 1–23. doi: 10.3389/fpsyg.2019.00990, PMID: 31316409 PMC6610331

[ref68] Weidinger A. F. Steinmayr R. Spinath B. (2017). Math grades and intrinsic motivation in elementary school: a longitudinal investigation of their association. Br. J. Educ. Psychol. 87, 187–204. doi: 10.1111/bjep.12143, PMID: 28155221

[ref69] Xiao F. Sun L. (2021). Students’ motivation and affection profiles and their relation to mathematics achievement, persistence, and behaviors. Front. Psychol. 11:533593. doi: 10.3389/fpsyg.2020.533593, PMID: 33519570 PMC7841336

[ref70] Yáñez-Marquina L. Villardón-Gallego L. (2017). Math anxiety, a hierarchical construct: development and validation of the scale for assessing math anxiety in secondary education. Anxiety Stress 23, 59–65. doi: 10.1016/j.anyes.2017.10.001

